# Thoracic Wall Reconstruction with Dorsal Diaphragmatic Traction and Preservation of Diaphragmatic Attachments in a Dog with Resection of the 11–13th Ribs

**DOI:** 10.3390/ani13010034

**Published:** 2022-12-21

**Authors:** Eiichi Kanai, Ayano Kudo, Asaka Sato, Hiromitsu Yoshida, Akinori Yamauchi, Ryo Oshita, Satoshi Takagi

**Affiliations:** 1 Laboratory of Small Animal Surgery, Azabu University, Kanagawa 252-5201, Japan; 2 Service of Soft Tissue Surgery & Surgical Oncology, Azabu University Veterinary Teaching Hospital, Kanagawa 252-5201, Japan

**Keywords:** thoracic wall reconstruction, diaphragmatic traction, thoracic wall resection, thoracic wall defect, thoracic wall tumor

## Abstract

**Simple Summary:**

This report describes a novel procedure for thoracic wall reconstruction. The thoracic wall defect caused by a tumor resection was successfully closed with the direct traction of the part of the diaphragm located directly underneath the defect and sutured to the ribs and thoracic wall muscles surrounding the defect. Complications typically associated with diaphragmatic traction, including respiratory distress and exercise intolerance, were not observed immediately after the surgery and during 190 days postoperatively. This technique for thoracic wall reconstruction is suggested to simplify the surgical procedure and it can safely be performed in small-size dogs weighing <5 kg.

**Abstract:**

A 9-year-old, 4.7 kg, spayed female Chihuahua presented with a 3.5 cm soft tissue sarcoma on the dorsal right thoracic wall. The tumor was resected, including the 11–13th ribs, resulting in a caudal dorsal thoracic wall defect. The defect was reconstructed with direct traction of part of the diaphragm dorsally, preserving the diaphragmatic attachments to the body wall, and the diaphragm was sutured to the surrounding ribs and muscles. Possible respiratory complications, including paradoxical respiration and exercise intolerance, were not observed during the perioperative or postoperative observation periods. This novel procedure is expected to be an option for caudal thoracic wall reconstruction when the diaphragmatic attachments remain intact even after the resection of the last rib. In addition, this procedure can be performed in dogs weighing <5 kg, with small pleural cavities and without respiratory disorders.

## 1. Introduction

Resection of thoracic wall tumors, including the ribs, generally requires reconstructive surgery for closure of the thoracic wall, using various techniques. The reconstruction of the thoracic wall must re-establish the airtightness of the pleural cavity, stabilize the thoracic wall, and protect the thoracic organs [1,2]. Some techniques for canine thoracic wall reconstruction have been reported, including prosthetic meshes [2,3,4,5,6] and flaps using the autogenous latissimus dorsi muscle [2,3,4,5], external abdominal oblique muscle [1], and omental pedicle [2,4,5]. A caudal thoracic wall defect can also be reconstructed using the free edge of the diaphragm advanced and sutured to the thoracic wall [3,4,5,7]. The choice of procedure for thoracic wall reconstruction depends on the size and location of the defect and whether an infection is present. Prosthetic materials cannot be used in infected wounds, and postoperative scarring and infection may require implant removal [2]. Reconstruction using an autologous muscle flap may limit the extent of flap motion and cover, and the flap may become necrotic [2]. Diaphragm advancement is indicated for reconstruction of defects between the 8th and 13th ribs, though it requires additional reconstruction of the abdominal wall [3,4,5,7].

A novel procedure for thoracic wall reconstruction has been reported, with traction of the diaphragm sutured to the thoracic wall when its body wall attachments are preserved [8,9]. This technique has been successfully performed only in two large breed dogs weighing >25 kg, thus the outcome in small dog breeds is still unknown. This case study describes a thoracic wall reconstruction with diaphragmatic traction after the resection of the 11–13th ribs in a dog weighing <5 kg.

## 2. Case Description

**Figure 1:**
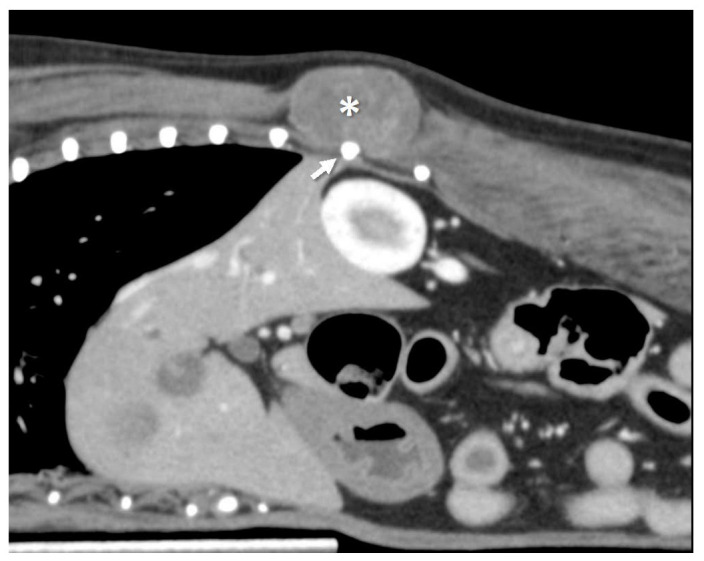
Contrast-enhanced CT image, sagittal plane. The tumor is adjacent to the 12th rib. The dog was positioned ventrally, with the cranial side on the left side of the image. The * indicates the tumor; the arrow indicates the 12th rib.

A 9-year-old, 4.7 kg, spayed female Chihuahua presented to the referring hospital for evaluation of a right-sided thoracic wall tumor. The tumor was diagnosed as a soft tissue sarcoma after a needle biopsy at the referral hospital. The dog was referred to Azabu University Veterinary Teaching Hospital for surgical resection of the tumor. On physical examination, the tumor was subcutaneous, soft, fixed at the base of the tumor, and immobile, and the patient was averse to having the lesion touched. No other anomalous findings were noted. The complete blood count and serum chemistry were within the reference limits. Computed tomography (CT) scan was performed to evaluate the tumor invasiveness and metastases to other organs. Then the tumor was situated in the sublayer of the latissimus dorsi muscle in proximity to the 12th rib, longissimus muscle, and intercostal muscles (Figure 1). The tumor size was 3.5 × 2.2 × 3.1 cm (length × height × width). The mean CT density of the tumor was 35 HU on plain images and enhanced to 69 HU on contrast-enhanced images. There was no evidence of metastasis to the lung or lymph nodes. Surgical resection of the tumor was planned based on the CT images. The horizontal margin was set at 2 cm from the edge of the tumor, and the deep margin was set at the full thickness of the thoracic wall, including the 11–13th ribs.

The dog was premedicated with atropine (0.025 mg/kg, subcutaneous [SC]) and fentanyl (5 µg/kg, intravenous [IV]), then induced with propofol (1.6 mg/kg, IV), intubated, and maintained with isoflurane in oxygen. Respiratory management was performed using a volume control ventilator set at a tidal volume of 68–80 mL and a respiratory rate of 15 cycles per minute. Pain management was performed with intercostal nerve blockade with 1 mL of 0.5% bupivacaine hydrochloride solution and continuous rate infusion (CRI) of fentanyl 5–10 µg/kg/h and ketamine 0.6 mg/kg/h. Preoperatively, prophylactic antibiotics were administered (cefazolin sodium, 25 mg/kg, IV). The intraoperative infusion was Ringer’s lactate solution at 5 mL/kg/h.

**Figure 2:**
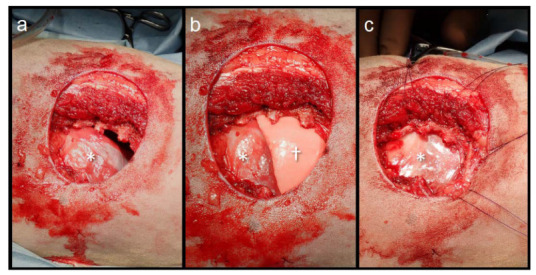
Images of the thoracic wall defect. (**a**) Full thickness of the thoracic wall resection, including the 11–13th ribs; (**b**) The lungs were inflated at 15 mm Hg on the pressure gauge of the anesthesia machine. The caudal border of the right caudal lung lobe advanced to approximately half of the defect site; (**c**) The diaphragm was pulled and sutured to the thoracic wall. The dog was positioned ventrally, with the cranial side on the right side of the images. *: diaphragm; †: right caudal lung lobe.

The dog was positioned ventrally. The tumor and full thickness of the thoracic wall, including the 11th, 12th, and 13th ribs, were resected en bloc with the scheduled surgical margins (Figure 2a). After removing the tumor, the diaphragmatic attachment to the body wall was preserved. The lung was inflated to a pressure of 15 mmHg with an air gauge from the anesthesia machine, and the caudal border of the right caudal lung lobe was advanced to approximately half of the thoracic wall defect site (Figure 2b). Before the thoracic wall reconstruction, an 8-Fr feeding tube was placed in the eighth intercostal space as the thoracotomy tube.

Primary closure of the thoracic wall muscles was attempted; however, the tension on the suture line was strong, and there was concern about the risk of wound dehiscence. The thoracic wall defect was reconstructed using dorsal traction of the diaphragm directly underneath the defect. The phrenic nerve and vessels were not identified at the diaphragm traction site. A 3-0 polypropylene suture (PROLENE, Ethicon, Raritan, NJ, USA) was used as a stay suture to pull the diaphragm. The diaphragm was drawn dorsally to reduce the tension on it and prevent circulatory failure due to kinking of the caudal vena cava. It was then sutured to the ribs and intercostal muscles at the edges of the thoracic wall defect using 3-0 polydioxanone sutures (MonoPlus, B. Braun, Melsungen, Germany) with horizontal mattress and simple continuous sutures (Figure 2c). The thoracotomy tube was aspirated and negative pressure in the pleural cavity was confirmed. The longissimus dorsi, longissimus, and external abdominal oblique muscles were closed with continuous sutures using 3-0 monofilament polydioxanone. The thoracic wall defect was reconstructed with two layers of traction of the diaphragm and muscles. The subcutaneous tissue was closed using 3-0 monofilament polyglyconate sutures (Monosyn, B. Braun) and the skin using 3-0 monofilament nylon sutures (Bearlon, BEAR Medic Corporation, Tokyo, Japan).

**Figure 3:**
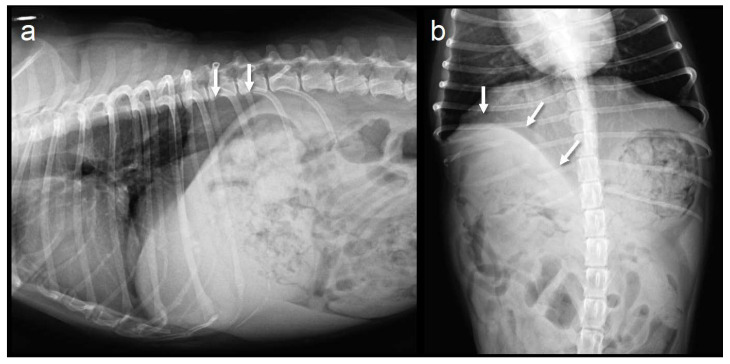
Abdominal radiographs at the 6-month follow-up. The suture line of the diaphragm and thoracic wall is visualized (arrows). (**a**) Lateral view; (**b**) Ventrodorsal view.

The dog awoke from anesthesia uneventfully. Postoperative analgesia consisted of fentanyl 2–3 µg/kg/h and ketamine 0.2 mg/kg/h CRI for two days postoperatively, and robenacoxib 1 mg/kg SC to the fifth postoperative day. The thoracotomy tube was removed within 24 h after the surgery, as no pneumothorax or pleural effusion was observed. Respiratory compromise, paradoxical respiration, or flail chest was not observed postoperatively. The dog was discharged on the fifth postoperative day. The tumor was diagnosed as a malignant peripheral nerve sheath tumor based on the histopathological evaluation, and complete resection was confirmed at the resection margins. At the 6-month follow-up (190 days postoperatively), there were no clinical signs related to diaphragmatic traction, and exercise intolerance was not observed in daily life in contrast to the preoperative conditions. Thoracic radiography revealed a line of the diaphragm sutured to the thoracic wall (Figure 3). There was no evidence of local recurrence or metastasis of the tumor.

## 3. Discussion

Canine thoracic wall defects are typically reconstructed using prosthetic materials, autologous muscles, or the diaphragm [1,2]. Prosthetic materials include polypropylene, polytetrafluoroethylene, and polyglactin [1,2,3,4,5,6,10,11] and are feasible for the closure of large areas of defects. However, postoperative persistence of infection requires surgical removal of the prosthetic material [2]. On the other hand, muscle flaps require covering the defect with minimal invasion, movement, and muscle tension [1], and a muscle flap may become necrotic due to impaired blood flow or persistent hematomas [2]. Diaphragm advancement is indicated for the reconstruction of thoracic wall defects associated with caudal rib resection (8–13th ribs) [3,4,5,7]. The defect is closed with the free edge of the diaphragm sutured to the cranial thoracic wall or the ribs. Abdominal wall defects resulting from diaphragmatic advancement must be closed with a muscle flap or prosthetic mesh if primary closure is not possible. In this case, the thoracic wall reconstruction after resection of the 11–13th ribs was planned with diaphragmatic advancement. The last rib was resected; however, the diaphragmatic attachment was preserved because the resection site was dorsal. The diaphragm was drawn dorsally, and it was possible to cover the defect adequately without excessive tension. Diaphragmatic traction is simple because of the positive pressure in the pleural cavity. Although there was some concern about a kink in the caudal vena cava due to right-sided diaphragmatic traction, there was no change in the intraoperative anesthesia monitoring. After negative pressure was re-established in the pleural cavity, there were no kinks or venous stasis in the caudal vena cava on radiographic or ultrasonographic examinations of the thorax and abdomen.

Diaphragmatic traction has been shown to affect the respiratory function due to decreased motility of the diaphragm and thoracic wall or decreased respiratory volume due to a reduced pleural cavity. In this case, we were concerned about a postoperative reduced pleural cavity because of the dog’s smaller body size compared to the two dogs previously reported undergoing a similar surgery [8,9]. Respiratory dysfunction was not observed during intraoperative anesthesia monitoring or postoperative blood gas analysis. As in previous reports, no clinical signs of respiratory dysfunction or exercise intolerance emerged in the postoperative daily life. There are potential spaces between the diaphragm and caudal thoracic wall, called the costodiaphragmatic recess and the lumbodiaphragmatic recesses, which the lungs may enter and exit with inspiration and expiration [12]. These spaces are presumably affected by various factors, including abdominal pressure and body position, and typically the lungs do not penetrate them, even at maximal inspiration. When the lungs were inflated intraoperatively, they expanded to half of the defect. The area not involved in the lung expansion may become a potential space. If the pleural space lost by diaphragmatic traction is the potential space, it is suggested that the decrease in the pleural cavity required for lung expansion is kept to a minimum to avoid adversely affecting the respiration. In the future, it must be considered that the loss of this potential space may reduce the reserve when dogs with diaphragmatic traction present with respiratory symptoms. The long-term effects of diaphragmatic traction require further follow-up as diaphragmatic motility has not been evaluated.

In this case, primary closure of the thoracic wall muscles was attempted; however, the tension on the suture line was strong, and there was concern about the risk of wound dehiscence. After diaphragmatic traction, the thoracic wall muscles were primarily closed. Supposedly, diaphragmatic traction distributes the tension on the thoracic wall and consequently reduces the tension on the muscular suture line. In addition, closing the thoracic wall with two layers, the diaphragm and muscularis, is important to achieve the goals of thoracic wall reconstruction: re-establishment of airtightness in the pleural cavity, stabilization of the thoracic wall, and protection of the thoracic organs.

## 4. Conclusions

Diaphragmatic traction may be considered an alternative thoracic wall reconstruction procedure in caudal rib resection with preservation of diaphragmatic attachments. This procedure may also be indicated in dogs weighing <5 kg with small pleural cavities.

## Data Availability

Not applicable.
